# Promoting Exercise and Healthy Diet Among Primary Care Patients: Feasibility, Preliminary Outcomes, and Lessons Learned From a Pilot Trial With High Intensity Interval Exercise

**DOI:** 10.3389/fspor.2021.690243

**Published:** 2021-07-16

**Authors:** Abbie E. Smith-Ryan, Mark A. Weaver, Anthony J. Viera, Morris Weinberger, Malia N.M. Blue, Katie R. Hirsch

**Affiliations:** ^1^Department of Exercise and Sport Science, Applied Physiology Laboratory, University of North Carolina, Chapel Hill, NC, United States; ^2^Human Movement Science Curriculum, Department of Allied Health Science, University of North Carolina, Chapel Hill, NC, United States; ^3^Department of Medicine and Biostatistics, University of North Carolina, Chapel Hill, NC, United States; ^4^Department of Community and Family Medicine, Duke University, Duke, NC, United States; ^5^Department of Health Policy and Management, Gillings School of Global Public Health, University of North Carolina, Chapel Hill, NC, United States

**Keywords:** cardiovascular health and disease, high intensity interval training, exercise is medicine, lifestyle intervation, pilot and feasibility study

## Abstract

Physical activity and healthy diet are recognized as effective approaches for disease prevention. Controlled laboratory clinical trials support these approaches, yet minimal data exists supporting implementation of exercise as medicine within a healthcare setting.

**Objectives:** To understand perception and barriers to exercise and nutrition from patients and physicians from a family medicine clinic (FMC) to inform the implementation of a laboratory-based exercise and nutrition lifestyle intervention (Phase I), and to determine the feasibility, adherence, and preliminary outcomes of implementing this lifestyle intervention into a FMC (Phase II).

**Methods:** In phase I 10 patients and 5 physicians were interviewed regarding perceptions of exercise and nutrition practices. In phase II patients at risk for cardiovascular disease were enrolled into a lifestyle intervention (*n* = 16), within a FMC, manipulating diet and exercise. Cardiorespiratory fitness (CRF), body composition, and metabolic blood markers were completed at baseline, after the 12-week intervention, and at 24 weeks. Feasibility was defined by patients who completed the intervention and number of sessions vs. total available.

**Results:** Prescribing high-intensity interval training and a meal replacement for 12 weeks in patients with at least one risk factor for cardiovascular disease, was shown to have moderate feasibility with 62.5% (*n* = 10) for patients completing the 12 week intervention, and poor feasibility for assessing effects 12 weeks after cessation of the intervention, with 50% (*n* = 5) participants returning. Tracking exercise electronically via FitBit had moderate fidelity (*n* = 9), with hardcopy logs yielding poor compliance (*n* = 6). This pilot study demonstrated preliminary effectiveness of this home-based approach for improving cardiorespiratory fitness with an average 4.31 ± 5.67 ml·kg·min^−1^ increase in peak oxygen consumption. Blood triglycerides and insulin were improved in 70% and 60% of the patients, respectively.

**Conclusions:** Despite moderate feasibility, a home-based exercise and nutrition has the potential to be used as an effective approach for managing and mitigating cardiovascular disease risk factors. There were key lessons learned which will help to develop and adapt a larger scale lifestyle intervention into a clinical setting.

**Clinical Trial Registration:** https://clinicaltrials.gov/ct2/show/study/NCT02482922, identifier NCT02482922.

## Introduction

Cardiovascular disease (CVD) is the leading cause of mortality in the United States and is responsible for 17% of national healthcare expenditures (Heidenreich et al., 2011). At the current rate, an estimated 40% of U.S. adults will develop CVD by 2030 (Heidenreich et al., 2011). CVD is largely preventable by practicing healthy lifestyle behaviors. Physical activity and healthy diet are widely recognized as effective methods for primary and secondary prevention for a number of chronic diseases, including CVD (Eyre et al., 2004), most notably by improving cardiorespiratory fitness (CRF) (Blair et al., 1989).

Despite the consensus regarding the importance of physical activity, most adults fail to meet even the minimum physical activity guidelines. Numerous studies have shown that lack of time is the most common cited reason for not exercising (Godin, 1994; Booth et al., 2000). High-intensity interval training (HIIT) is a time-efficient exercise strategy that uses short, intense exercise periods interspersed with recovery periods. HIIT provides multiple advantages over traditional aerobic exercise including reduced time demand and equipment needs. HIIT has had no intervention-related adverse events reported, with reportedly high tolerability in a variety of clinical populations including patients with heart failure (Wisløff et al., 2007), stroke (Askim et al., 2014), and cancer (Persoon et al., 2010; Wood et al., 2016). Additionally, physiological benefits from HIIT appear to be equal to or greater than traditional aerobic exercise, but achieved in a shorter time frame (Helgerud et al., 2007; Nybo et al., 2010). Beyond HIIT, *ad libitum* meal replacement is second only to bariatric surgery as an effective way to elicit weight loss (Casazza et al., 2013). Meal replacements have been shown to reduce obesity (Casazza et al., 2013), and could be combined with HIIT as a practical strategy for reducing CVD risk as a result of the concomitant comorbidities. Recent evidence suggests that portioned-controlled, nutrient dense meal replacements may be one of the most effective strategies for weight loss (Lockwood et al., 2008; Casazza et al., 2013), as their use minimizes drastic behavior modification, simplifies decision making, and results in greater long-term compliance (Levy and Heaton, 1993; Heymsfield et al., 2003).

While a large evidence base demonstrates the efficacy of highly-controlled, laboratory-based exercise regimens such as HIIT (Campbell et al., 2019; Martland et al., 2020), translating these interventions into practice remains largely unexplored. A recent publication by Gray et al. (2016) urged for “real-world effectiveness” studies that evaluate lifestyle interventions outside of a controlled laboratory setting to facilitate more widespread use. One such setting for delivering home-based exercise and nutrition programs may be a primary care setting, or more commonly described as a patient-centered medical home (PCMH), which is the dominant primary care delivery model in the United States in which patient care is individualized and patient-focused (Hoff et al., 2012). A PCMH is a team-based health care delivery model where comprehensive patient treatment is coordinated through their primary care physician (O'dell, 2016). Prescribing exercise and nutrition as medicine within a primary care setting/PCMH has the potential to foster delivery and implementation of a home-based lifestyle program; we are unaware of such a program being implemented or published. Therefore, the purpose of this study was two-fold: (1) to qualitatively solicit feedback from patients and physicians from a PCMH (Family Medicine Clinic) to understand their experiences and barriers to an exercise and nutrition lifestyle intervention (Phase I) and (2) to determine the feasibility, adherence, and preliminary outcomes of an exercise and nutrition lifestyle intervention (Phase II).

## Materials and Methods

This single-arm pilot study was conducted in a certified level 3 PCMH Family Medicine Center (FMC) of a large, academic medical center in collaboration with medical personnel within the clinic. For Phase I 10 patients and five physicians were included, which was identified a prior for adequate them saturation. For Phase II, ~15 participants were needed to detect estimated changes in cardiorespiratory fitness. This was not powered directly for hypothesis testing and the laboratory based data was used to estimate sample size for this pilot trial. Inclusion/exclusion criteria of participants were the same for both Phase I and Phase II: (1) 18–85 years of age; (2) received primary care in the FMC (≥ 1 visit during the previous year); and (3) had ≥ 1 risk factor for CVD (blood pressure > 130/85 mmHg; BMI > 25 kg/m^2^, fasting glucose > 110 mg/dl, high density lipoproteins (HDL <30). Participants were excluded if diet and exercise were contraindicated, or if they were at risk of death in the next year (e.g. Class IV heart failure, end-stage renal disease), both of which were determined by their primary care provider. Patients were also excluded if they had been taking medication for diabetes, blood pressure, or lipids for more than two years; were pregnant or planning on becoming pregnant; or their primary care provider did not believe they should participate. Additionally, patients were excluded if they did not have access to a computer or smartphone due to the use of an activity monitor. All procedures were approved by the University's Biomedical Institutional Review Board.

### Program Refinement (Phase I)

The HIIT exercise program and meal replacement approaches were based on previously published laboratory based interventions that had demonstrated to significantly improve health parameters (Lockwood et al., 2008; Smith-Ryan et al., 2015, 2016). A phone interview was implemented in order to gather broad perspective from patients and physicians regarding preferences for the type of exercise and nutrition strategies that were preferred, and to understand if there were any major changes that needed to be made to the laboratory-based program before implementing into a clinic. Patients (*n* = 10) participated in one semi-structured telephone interview during which the goals of the lifestyle program were described, and a series of open-ended questions were asked (Table 1A); all patients interviewed were currently seeking care at the same FMC that was used for the intervention; patients were eligible to participate in Phase II, but none of them choose to. Recruitment for Phase II occurred several months after Phase I and used in person flyers; Phase I participants were not contacted directly. Physicians that were interviewed and consented (*n* = 5) included personnel within the FMC, as well as physicians at the same institution that had a complementary medicine background or had a public health care focus; physicians were interviewed via telephone and were engaged in a series of open-ended questions (Table 1B). The interviews were not meant to be qualitatively analyzed for statistical theme saturation, but instead used to tweak recruitment strategies, understand the climate of the clinic with regard to using an exercise as medicine approach, and guide conversations with patients and physicians.

**Table 1:** Patients (*n* = 10) and Physicians (*n* = 5) interview questions.

**1A. Exercise Related Topics**
(1) Previous exercise experience (type of exercise, consistency and recent history of exercise)
(2) Preferred structure of an exercise program (mode of exercise, environment, duration, intensity)
(3) Preferred delivery of an exercise program (one-on-one, group setting, in-person, videos, etc.)
(4) Facilitators and barriers toward participating in exercise
Nutrition Related Topics
(1) Previous experience with healthy eating
(2) Supplementation use
(3) Primary source for nutrition information
(4) Facilitators and barriers to healthy eating
**1B. Physician Discussion Points**
- Current referral/recommendation practices for exercise and nutrition for patients
- Common barriers to exercise and healthy lifestyle practices among patients
- Opinions on improving adherence and feasibility of prescribing exercise and nutrition as a preventative health strategy
- Clinical perspective on the use of higher intensity exercise for their patients
- Opinion on nutritional recommendations and meal replacements as a nutritional intervention
- Primary source for nutrition information

### Feasibility and Pilot Intervention (Phase II)

#### Procedures

Using a single group, prospective pilot approach, patients were recruited and consented by the research team through: (1) flyers posted within the FMC; (2) review of medical records for eligible participants with a limited HIPAA waiver; or (3) referral from nurses and physicians within the clinic. Physiological measurements of body composition, cardiorespiratory fitness, and fasting blood variables were taken before the intervention (baseline), after the 12-week intervention (3 months), and 12 weeks after the intervention ended (6 months). All measurements and interaction with participants were completed in a FMC exam room using portable equipment implemented by the research team. All results were integrated into the patient's medical record, including cardiorespiratory fitness, body composition, and blood results; communication occurred initially with the participant's primary care physician for inclusion clearance and study details; communication was maintained through electronic medical record with their patient-physician team.

#### Home-Based Lifestyle Intervention

The baseline HIIT program was developed and validated from a laboratory-based protocol (Smith-Ryan et al., 2015, 2016) and relied on maximal heart rate (HR) obtained during cardiorespiratory fitness (VO_2_peak) testing to establish individualized training intensities. During baseline testing at the FMC, patients were individually counseled by the exercise physiology research staff who discussed local training resources that were available and adaptable to individual participants' needs and preferences (e.g., walking, jogging, cycling, stationary exercise equipment, stairs). Participants were instructed to complete a five-min warm up, followed by 10 sets of one-min intervals at 75–95% maximal HR, with one-min active or passive rest between sets. In order to track intensity, participants were provided with a Polar Heart Rate Monitor (Model FT1, Warminister, PA, USA) to track HR during all exercise sessions. Additionally, all patients were asked to wear a FitBit wrist activity tracker (FitBit Charge HR, San Francisco, CA) every day for the duration of the intervention, and were asked to intentionally track the exercise session (Diaz et al., 2015; Rosenberg et al., 2016). Traditional chest-strap HR monitoring is accepted as a more accurate method (Gillinov et al., 2017), but requires a chest strap and puts more ownness on the subject to review and remember each interval on the watch for HR values and then record them on a paper log, thus both methods were included to enhance tracking, compliance, and feasibility. Participants were instructed to complete the HIIT program 2 times per week, with 3 times per week encouraged; twice weekly sessions was used for compliance evaluation. At the baseline appointment, all patients received in-depth instructions on how to use the heart rate monitor and the FitBit; the research staff also set up the FitBit on participants' phones and initiated the monitoring. At this visit, participants also received a paper calendar document that covered the dates of their participation, and were asked to manually track when they completed the exercise, their maximum HR they obtained, number of intervals, and any qualitative feedback. In addition to the technological monitoring, patients also received bi-weekly phone calls and emails from the research team to check in and help troubleshoot both the technology and any questions related to the HIIT.

**Figure 1:**
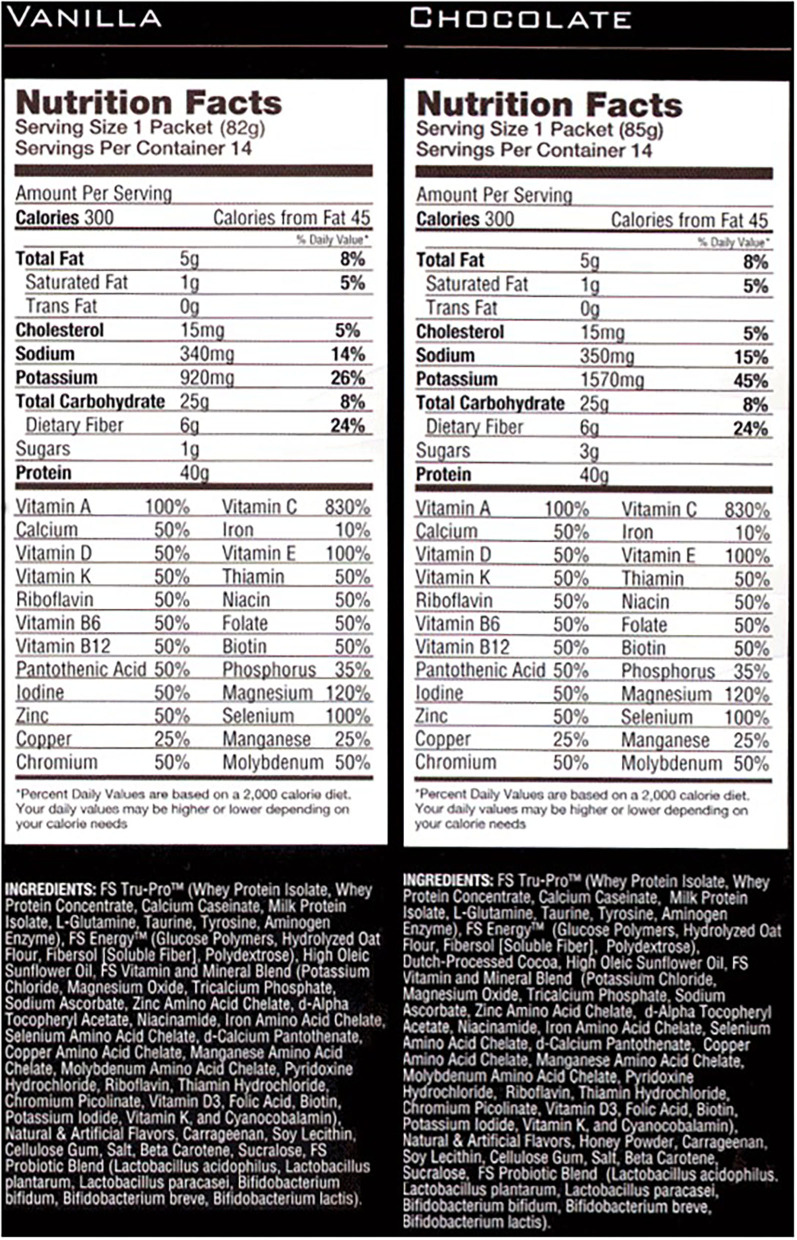
Meal replacement supplement facts panel.

In addition to the HIIT program, the research team provided patients with meal replacement shake and were asked to consume it once daily *ad libitum*. The intention of the meal replacement was not to replace a specific meal, but to naturally modify the total daily dietary quality. The meal replacement was packaged in individual single serve packets and was high in complex carbohydrates, protein, and fiber (Full Strength®, Phillips Performance Nutrition, LLC, Golden, CO) (Figure 1), and previously demonstrated improvements in body composition and blood lipid profiles (Lockwood et al., 2008). Participants were provided a shaker cup, were instructed to mix the shake in accordance with the label directions, and were otherwise instructed to maintain their current diet.

### Outcomes of Feasibility and Compliance

Feasibility of the program was determined from the screening and enrollment process, as well as through the fidelity of participants at the 3 month and 6 month follow-ups. Compliance for the HIIT program was defined by total number of sessions completed/recorded out of total number available (24 sessions). FitBit data was also used to support compliance for tracking sessions and step counts. Compliance for the meal replacement shake was determined by counting the empty packets; participants returned the empty packages upon return to baseline, as well as cross-reference with hard copy log.

### Outcomes to Assess Clinical Effectiveness

#### Body Composition

After a minimum of a six-h fast, percent body fat (%Fat), fat mass (FM), and fat-free mass (FFM) were evaluated using two methods of portable composition assessments: bioelectrical impedance spectroscopy (BIS; SFB7 Impedimed, Queensland, Australia) and amplitude mode ultrasound (A-Mode US; BX-2000, IntelaMetrix, Livermore, CA). Age, height, weight, and sex were entered into each system. For BIS measurements, after five min of supine rest, two single-tab electrodes were placed 5 cm apart on the subject's right wrist and hand, and two were placed on the right ankle and foot. The average of the two trials were used for final values.

For A-mode US measurements, subcutaneous adipose tissue thickness was measured using a standard 2.5 MHz probe. The probe was connected by USB to a standard laptop with corresponding proprietary software (BodyView Professional Software) (Smith-Ryan et al., 2014). Measurements were taken on the right side of the body, while the subject was standing using seven-site skin-fold locations according to Jackson and Pollock (Jackson and Pollock, 1978; Jackson et al., 1980). The measurement sites included chest, triceps, subscapular, suprailiac, midaxillary, abdomen, and thigh. Measurements were made by liberally applying transmission gel to the probe and lightly placing the probe perpendicular to the site. Each site was measured two to three times, based upon the software's agreement between measurements, and the average of these trials were used to represent the final thickness measurement. For both devices, FM and FFM were calculated as follows: %body fat × body mass = FM; Body mass-FM = FFM.

#### Cardiorespiratory Fitness

Using a modified graded exercise test on a cycle ergometer (Star Trac S-UBx, Vancouver, WA), maximal HR was captured in order to establish individual training intensities for the HIIT program, as well as assess peak oxygen consumption (VO_2_peak). Before beginning the test, patients were fitted with a heart rate monitor (Polar FS1, Polar Electro Inc. Lake Success, NY) and the bike seat was adjusted for comfort. A facemask was secured to the patient with fabric headgear and connected to a portable metabolic system (Fitmate Pro, Cosmed, Rome, Italy), which was used to indirectly analyze oxygen consumption. After a three-min warm-up, the workload increased by 20 watts every min until the patient could no longer continue despite verbal encouragement. The highest value was recorded as VO_2_peak.

#### Fasting Blood Variables

Serum blood samples were drawn following a minimum of an 8 h fast, and were drawn by clinic phlebotomy staff and were separated and processed by McLendon Clinical Laboratories (Chapel Hill, NC). All samples were analyzed using established enzymatic assays for fasting blood glucose, total cholesterol, HDL, low density lipoproteins (LDL), triglycerides, and hemoglobin A1c. Insulin was shipped and analyzed by Mayo Clinic Laboratories (Rochester MN), according to routine clinic procedures.

#### Data Analysis

A qualitative description for Phase I is presented displaying common themes and selected quotations. For phase II primary outcomes, feasibility data were summarized using recruitment and retention rates, and exercise sessions completed. For clinical effectiveness, as recommended by CONSORT, estimates of change over time with 95% confidence intervals were reported and formal hypothesis testing was not completed. Ninety-five percent confidence intervals were determined on change scores calculated from linear mixed models to account for paired data. Mean change in outcomes from baseline to three months were reported for systolic blood pressure (SBP), diastolic blood pressure (DBP), %Fat, FM, LM, abdominal fat, relative VO_2_peak, max HR, and a series of blood markers. Normality of the data was assessed and confirmed. Means and standard deviations (SD) are presented to describe the sample. Statistical analyses were performed using SAS, version 9.3 (SAS Institute, Cary, NC).

## Results

### Program Development and Refinement (Phase I)

In Phase I, ten patients (mean ± SD; Age: 43.6 ± 8.8 yrs; Height: 174.8 ± 8.5 cm; Weight: 100.5 ± 34.7 kg; BMI: 32.6 ± 9.2 kg/m^2^) and five physicians participated.

#### Patient Interviews

Patients reported minimal engagement in exercise; when participation in exercise was noted, activities included walking or elliptical use, all at a low-intensity steady state pace.

“*I currently try to walk for exercise.”*

“*I would be interested in high intensity training if someone showed me how to do it.”*

Patient preferences regarding mode and delivery of exercise varied, with a desire for walking and non-impact options. Additionally, patients generally desired accountability and flexibility in their exercise programs.

“*I enjoy the social aspect of exercise and having some accountability.”*

Commonly mentioned facilitators included weight loss, increased energy and productivity, doctor's recommendation, or a health diagnosis from their physician. Common barriers included lack of time/schedule, pain and risk of injury, resources/equipment availability, and the process of getting started.

“*I am motivated to lose weight”*

“*I am motivated for my children, to set a good example for them and to have more energy.”*“*There are too many benefits from exercise no to do it.”*

With regard to nutrition, patients' common concerns were about consuming large portion sizes, as well as skipping meals. Their knowledge of nutrition came primarily from the internet, family, and popular press readings. There was nearly no supplement use, except for an occasional multi-vitamin. Facilitators for healthy eating were focused on existing knowledge base, children, and weight management. Barriers centered on finances and lack of time and knowledge.

“*I know that I consume too much sugar and eat too much fried food.”*

“*Eating healthy is expensive.”*

#### Physician Interviews

Overall, physicians were enthusiastic about integrating exercise and nutrition into discussions with their patients, however, they all identified a lack of time during their visits with patients to adequately discuss these topics. The most common referral for lifestyle changes they made was to physical therapists, but only when the condition was chronic. Common barriers noted for their patients included knowledge/resources, time, cost, depression, and pain.

“*Resources can be an issue for exercise-the neighborhood is not always safe to exercise outside or do not have enough money to join a gym”*

“*For a program to be successful I believe it needs to meet the primary barriers of time and pain (i.e. arthritis).”*

Overall physicians felt as if high-intensity exercise was not appropriate for most of their patients due to obesity or health risk. To improve feasibility, incorporating at-home approaches to exercise with inclusion of feedback tied to their health and medical costs were suggested.

“*I worry that obese individual cannot sustain this type of exercise or may result in injury.”*

“*A diagnosis – such as diabetes or hypertension, is the best motivator for my patients to initiate a lifestyle change”*

“*The best approach is to teach the patients how to do the exercise on their own. Schedule it in an already scheduled visit*.

“*Outside reinforcement, someone that is not their clinician, that circles back around to the patient regarding their diagnosis can be helpful in the management of their disease.”*

Meal replacements and supplements were not typically recommended as an approach due to cost and opinions on efficacy.

“*Cost is a main concern for patients; I don't typically recommend supplements due to the additional cost.”*

Based on feedback and conversations from Phase I, a few programmatic changes to account for key barriers took place for Phase II. Changes to the program included planned communications with participants and physician's regarding their results; the number of sessions per week were reduced to 2, while encouraging a third; and weekly contacts were added. More specifically, documents were created to provide participants with all baseline and post-intervention health data, as well as information about what the data meant to them individually. This was done to emphasize the results directly to their health. Based on the accountability feedback, the FitBits were included as a means for the patients to track heart rate, exercise frequency, and progress. Additionally, in depth discussions of the flexibility of the training were included to describe it could be done with minimal equipment and few resources. Weekly emails or phone calls were included to also support accountability. Direct communication with their physician for patient participation and progress was included.

### Feasibility and Compliance Outcomes (Phase II)

**Figure 2:**
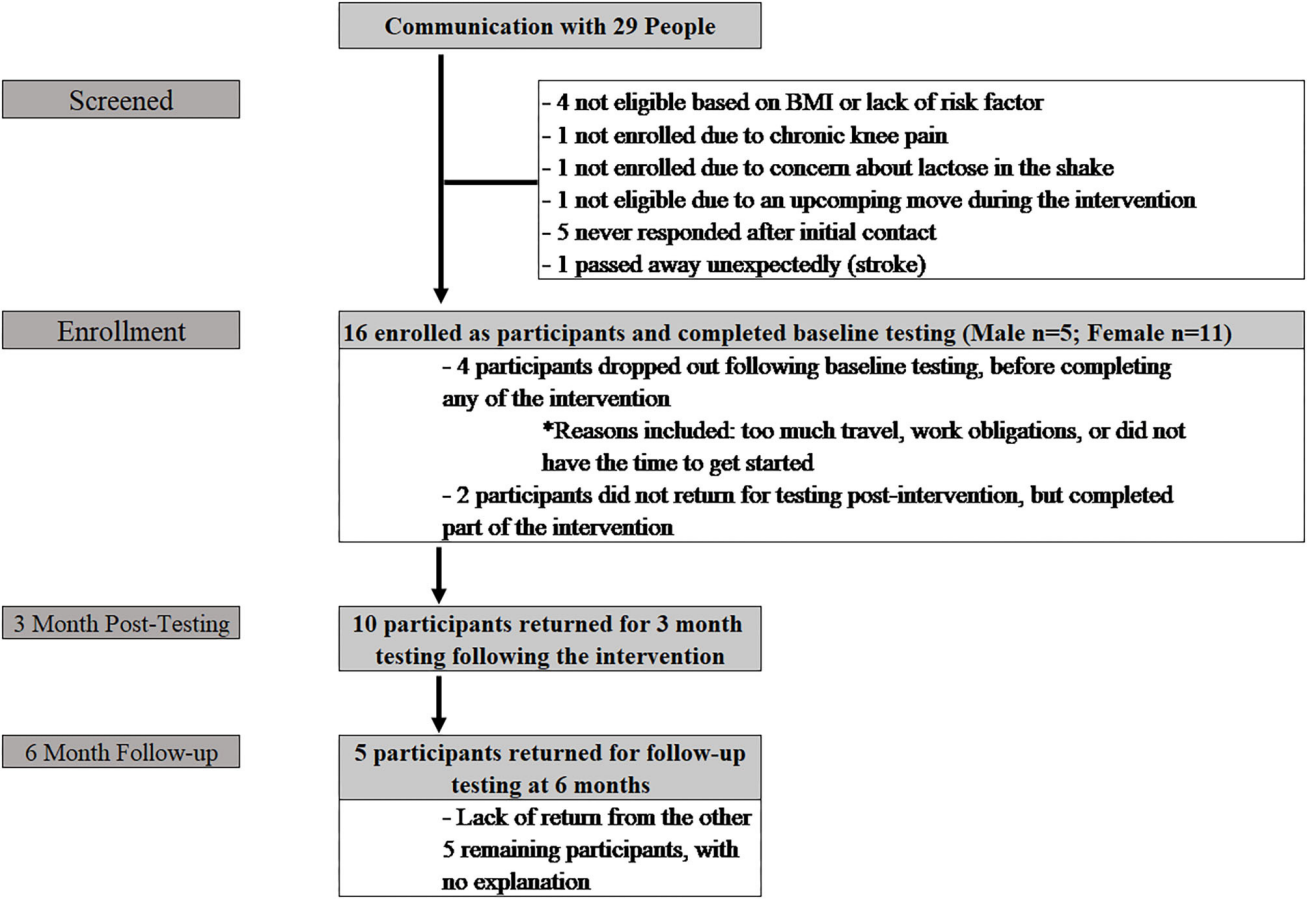
Details of participant recruitment, maintenance, and follow-up.

Phase II included initial contact with 29 people (Figure 2), 16 of whom enrolled (Age: 44.9 ± 12.6 yrs; Height: 168.6 ± 9.3 cm; Weight: 94.4 ± 18.2 kg; BMI: 33.1 ± 5.6 kg/m^2^). The additional 13 participants did not enroll for various reasons, including travel during the time of the intervention, not currently displaying a cardiovascular risk factor, no access to a computer/smartphone, living too far away from the clinic, and lack of response from initial contact. Six participants (37.5%) (Age: 35.2 ± 14.1 yrs; Height: 164.8 ± 8.7 cm; Weight: 89.8 ± 17.6 kg; BMI: 33.1 ± 6.5 kg/m^2^) completed baseline testing, but did not return for post-testing; four of these participants did not initiate any training; the remaining two participants dropped out within the first two weeks of the program. Of these six participants, five were male and one was female. Ten participants (62.5%) (Age: 50.8 ± 7.1 yrs; Height: 171.0 ± 9.3 cm; Weight: 97.2 ± 18.8 kg; BMI: 33.1 ± 5.4 kg/m^2^) returned following the intervention for post-intervention (3 month) assessments. Of those ten individuals, three were male and seven were female. Five participants (31%) returned for testing at the six-month follow-up (male = 2; female = 3).

Of the 10 participants who completed the duration of the 12-week lifestyle program, nine utilized the FitBit for some aspect of daily activity (i.e., activities of daily living) (Table 2). An average of 76.2 days (median 84 days) were recorded electronically. The average steps per day reported was 9,268 ± 2,397 steps, with a median of 8,665 steps. Direct compliance numbers for the HR chest strap were not captured, but participants did report using them often; however, the FitBit was preferred over the chest strap. Part of this was due to the additional watch band that was required for viewing HR. Only six participants returned hardcopy training logs; review of hard copy training logs yielded completion rates for a HIIT workout of 17%, 33%, 42%, 88%, 100%, and 100%, respectively. Adherence with the meal replacement was high with an average of 88% of the shakes being consumed. Bi-weekly phone calls that took place throughout the duration of the study included common issues and discussions related to trouble syncing fitbits, or not syncing units with their smartphone; conversations regarding options for completing the HIIT exercise; if they could complete more sessions than prescribed; how to proceed if they did not complete the recommended sessions. All issues were discussed and resolved with the PI or a member of the research team.

**Table 2:** Details of compliance for paper logs, FitBit and HR chest monitor for tracking exercise.

	**Subjects Used/Completed**	**Days/sessions recorded**
Paper Logs	6/10	4/24
		8/24
		10/24
		21/24
		24/24
		24/24
FitBit	9/10	76.2/84 total days
HR Chest Strap	Not tracked automatically; not captured.	

### Clinical Effectiveness Outcomes

#### Cardiovascular Risk Factors and Cardiorespiratory Fitness

**Figure 3:**
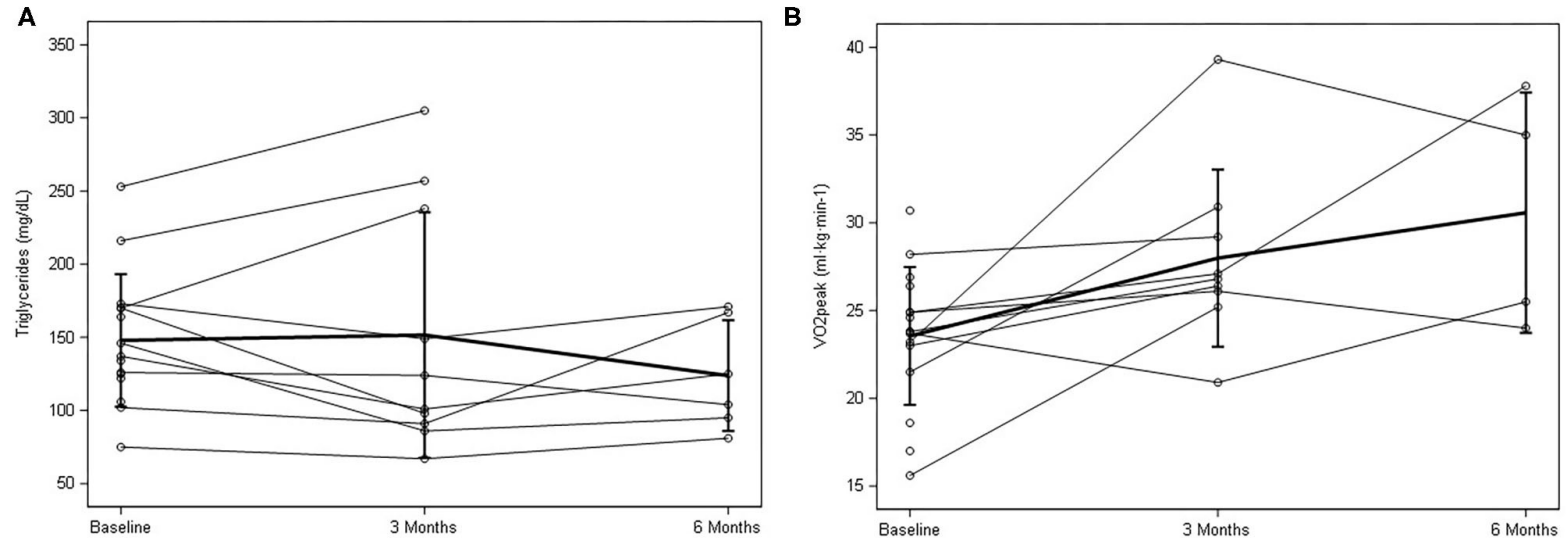
Individual response and mean ± SD data for baseline, 3 months, and 6 months for **(A)** triglycerides and **(B)** peak oxygen consumption (VO_2_peak.).

Following the at-home lifestyle program, 8 of 10 participants (80%) improved their cardiovascular risk profile by improving at least one of their risk factors (Table 3). Five participants improved overall blood pressure (both SBP and DBP) with an average 3.8 (±8.0 mmHg) decrease in SBP and 1.4 (±4.6 mmHg) decrease in DBP. For blood profiles, there was a general improvement in 50% of the participants. Cholesterol was the least affected, with 30–40% of participants improving in TC, HDL, and LDL collectively. Seventy percent of the participants improved in the TG profile (Figure 3A), 60% in insulin, and 50% in glucose. At the six-month follow-up, nearly all changes mimicked baseline or decreased. Regarding body fat percentage, overall changes were negligible, with three participants losing a clinically meaningful amount (>2.25%) of body fat at 12 weeks. There was also an average 3.4 (±14.4 cm) change in abdominal fat. For cardiorespiratory fitness, 80% of the participants improved VO_2_peak values, with an average increase of 4.31 ± 3.8 ml·kg·min^−1^ (Figure 3B).

**Table 3:** Change in physiological variables from pre to post-intervention (12-weeks) presented as mean ± SD (*n* = 10).

		**Mean Change (*n* = 10)**	**Confidence interval**
Average Systolic BP (mmHg)		−3.8 ± 8.0	(−13.0,5.5)
Average Diastolic BP (mmHg)		−1.4 ± 4.6	(−6.8,3.9)
Body Fat%			
	BIS (%)	0.5 ± 1.0	(−0.8,1.7)
	Ultrasound (%)	0.3 ± 0.8	(−0.7,1.3)
Abdominal Fat (cm)		−3.4 ± 14.4	(−20.0,13.2)
VO_2_peak (ml·kg·min^−1^)		4.8 ± 3.8^*^	(0.3,9.2)
Total Cholesterol (mg/dL)		4.1 ± 10.8	(−8.2,16.4)
Triglycerides (mg/dL)		−5.2 ± 29.6	(−38.6,28.2)
HDL (mg/dL)		1.4 ± 3.6	(−2.7,5.5)
LDL (mg/dL)		3.7 ± 15.6	(−14.0,21.4)
Glucose (mg/dL)		−8.0 ± 23.4	(−34.4,18.4)
Insulin (IU/L)		0.6 ± 10.0	(−12.2,11.0)
Hemoglobin A1c (mg/dL)		5.4 ± 10.2	(−16.9,6.2)

* *indicates significant change*.

## Discussion

While data regarding the efficacy of HIIT in various clinical populations is rapidly expanding, real-world translation of these findings is stifled by specialized equipment or very controlled prescription. This pilot study, to our knowledge, is the first to examine the feasibility and preliminary clinical effectiveness of a combined home-based HIIT and meal replacement intervention in conjunction with physician providers at a PCMH. In designing the lifestyle program, we integrated feedback from patients at risk for CVD and their primary care physicians. Implementation of the 12-week HIIT and meal replacement lifestyle program appeared to be moderately feasible with 10 out of 16 patients with at least one CVD risk factor returning for post-testing. Compliance to the program was variable with difficulty in tracking the workouts from both hard copy logs and electronic FitBit monitoring (average 64% logged compliance). Feasibility of patients returning 3 months after the intervention was complete (6 months), was low with only 50% returning (*n* = 5/10), which may have been influenced by the lack of contact during that time. There were no adverse events reported during the intervention. The program also appeared to elicit improvements in cardiorespiratory fitness (Δ + 4.8 ± 3.8 ml/kg/min^−1^) and triglycerides (Δ-5.2 ± 29.6 mg/dL). These data highlight some key considerations when performing a clinical trial within a medical clinic; it does appear to be a potentially feasible route for prescribing a lifestyle program, but dropout was higher (37.5%) than typically seen in our similar laboratory-based interventions. Tracking compliance was problematic; using an electronic method or an instantaneous way of tracking vigorous activity would be recommended. Despite the moderate adherence, significant improvements in CRF resulted which may support a future larger trial for implementing an exercise and nutrition program in a primary care setting. This data also supports the concept that a minimal intervention that initiates some exercise and change in nutrition, can have an impact on mitigating cardiovascular risk factors.

Input from patients and physicians was elicited to adapt an existing laboratory evidenced-based lifestyle intervention that consisted of thrice weekly cycling exercise in a one-on-one setting, including 10 bouts of 1 min high-intensity (~90%) interspersed with 1 min rest (Smith-Ryan et al., 2015, 2016). The laboratory-based program was modified to include various options for exercise that did not require equipment (i.e. walking/jogging, stair climbing) and variability in the days per week to complete the exercise (from 3 sessions to 2 sessions). To address patients' expressed desire for instruction and accountability, detailed verbal and written instructions were given at the initial visit, and bi-weekly phone calls or emails to address questions that arose. As technology has improved, an online delivery platform may be a beneficial tool for future implementation, as the bi-weekly phone calls/emails may be beyond the time value. Additionally, FitBits were included alongside a traditional heart rate monitor to allow participants to quickly see and receive feedback on workout details. A limitation of this approach was the inability to determine completion of actual number of intervals per session, which may influence future progression and adherence. The nutrition aspect of the program was developed based on previous data reported by Lockwood et al. (2008) reporting significant benefits of *ad libitum* consumption of a meal replacement on body composition and lipid profile. This approach also addressed feedback regarding portion sizes and convenience. The meal replacement appeared to be easy to use and adapt to, with high compliance.

### Feasibility

When implementing the program in Phase II, recruitment was similar to our laboratory based interventions, and was generally successful. Use of medical records for recruitment and communication with physician's for enrollment clearance was a streamlined approach, and would be recommended for a future larger trial. Based on the inclusion/exclusion criteria set in the present study, numerous patients were eligible. As with all exercise and nutrition studies, enrollment tends to target individuals that are motivated for change. Despite the successful screening and enrollment, dropout was higher than anticipated. Lack of time continues to be consistently associated with low physical activity levels (Trost et al., 2002; Korkiakangas et al., 2009). Despite the minimal time requirements of the current program, personal/business travel and lack of time were stated as primary reasons for discontinuing the program. Additionally, motivation and lack of group support may have played into the higher dropout. An interactive implementation or more virtual implementation may be more successful, which might more closely mimic the one-on-one laboratory based approach, which has very high compliance. In contrast, high adherence to the meal replacement was reported. Utilizing two flavor choices, chocolate and vanilla, over the three-month time span appeared to be helpful for participants, with all participants choosing a mix of both flavors.

### Clinical Effectiveness

High-intensity interval training has the potential to be promoted as a scalable at-home exercise program to delay progression and improve symptoms of type II diabetes (Babraj et al., 2009; Little et al., 2011), CVD (Weston et al., 2014), metabolic syndrome (Tjonna et al., 2008), and obesity (Weston et al., 2014; Smith-Ryan et al., 2015, 2016). One attribute of HIIT is the quick rate of response, with beneficial physiological effects occurring after as little as two weeks of training (Little et al., 2011). With rapid improvements in CRF and insulin sensitivity, exercise could feel easier, quicker which could be advantageous for overcoming common initial barriers to exercise related to discomfort or difficulty (Korkiakangas et al., 2009). Adoption of a similar HIIT approach has been shown to be feasible and effective when implemented in a group/community setting in overweight/obese individuals (Lunt et al., 2014). Previous data from Lunt et al. (2014) reported an average 0.6 ml·kg·min^−1^ change in VO_2_peak following thrice weekly of high-intensity training in a real-world group setting over 12 weeks. The volume and time of exercise of their protocol was much lower (3 × 30 s), which may explain the lower change in VO_2_peak compared to the 4.8 ± 3.8 ml·kg·min^−1^ change in the current study. Based on initial feedback, our participants may also have been more motivated due to the delivery of the program through a healthcare setting. Although not significant, the present study demonstrated a positive effect on triglycerides (70% improvement). This is consistent with previous data supporting significant improvements in triglycerides from 8 weeks of HIIT (Elmer et al., 2016), and from our previous data demonstrating non-significant, but clinically relevant improvements from 3 weeks of HIIT (Smith-Ryan et al., 2015). Changes in the blood profile may have also been influenced by the meal replacement, which was high in fiber. Previous data using the same shake combined with an exercise program, resulted in a significant improvements in lipid profile (TC, HDL, LDL, TG), but prescribed two shakes per day (Lockwood et al., 2008).

### Key Lessons Learned

Recruitment for eligible participants was similar to our laboratory-based interventions. Recruitment and enrollment were completed via medical record screening and with direct suppose from the physicians and clinic staff, which would be important for future clinic based implementation of a lifestyle intervention. Twelve of the 16 enrolled participants initiated the lifestyle program. Barriers for initiation centered on lack of time. The minimal time commitment (20 min, 2 x per week) appeared to overcome some of this, but motivation and other life priorities clearly influenced the tracking and testing of the intervention. To enhance successful enrollment for future similar interventions, a better explanation and emphasis on the minimal time required to see results with the intervention may be warranted. An additional in person contact early in the intervention may also help to prevent early dropout. A better understanding of initial goals of the participants and motivation for enrollment may be helpful to help tailor the program and align follow-up contacts with those goals. A qualitative assessment to understand characteristics of the subject, and their desire to participate, would be recommended to evaluate the participants that completed the intervention and those that chose to discontinue. Tracking adherence was another key lesson. While 10 of the 12 enrolled participants returned for post-testing, neither method of tracking (FitBit or hard copy logs) was well-received by participants. Only six participants returned the hard copy exercise logs, with missing entries on the latter half of the study; nine participants had FitBit data, but only four tracked the exercise sessions. Utilizing an activity monitor that automatically tracks intentional/higher intensity activity, or implementing an electronic training log, may be a better strategy. Additionally, while the technology was described in detail, a more intensive training on its use (including expectations for wearing and charging the device) may be helpful, or a virtual implementation. This type of tracking may also naturally exclude individuals that are uncomfortable with technology. Despite what appeared to be low tracking of exercise (63% adherence from available logs), CRF was significantly improved with twice weekly training over 12-weeks. However, sustaining these improvements after the training period may be challenging, as six-month values were similar to baseline; this may have also been influence by the low rate of return at 6 months. Providing a booster session after the program may improve adherence following cessation of the 12-week intervention. Providing a booster session after the program or reducing the intervention to once weekly for a longer duration may improve adherence. Lastly, while contacts were made bi-weekly, participants mentioned more contact would have supported accountability. Having an additional in-person or virtual session half-way through the program may have improved compliance (or tracking adherence). The meal replacement portion of the program was well-received, and compliance was high. Future programs may consider making other dietary changes and modifications in combination with a pre-packaged meal replacement.

## Conclusions

The present pilot and feasibility clinical trial revealed that an at-home lifestyle program for patients at risk for cardiovascular disease is feasible, safe, and may be effective in improving cardiorespiratory fitness. The increases demonstrated in cardiorespiratory fitness are an important outcome, due to the corresponding decrease in numerous associated comorbidities. Due to the potential benefits, a larger scale trial, incorporating a sufficient number of patients, along with an evaluation on the cost effectiveness, may provide insights for incorporating such a program in the PCMH or other clinical settings. Incorporating an electronic method for tracking compliance, as well as potentially including electronic messaging for reminders and goals, may be beneficial to include. Lastly, while this study was done in conjunction with a PCMH and recorded participation and data within patient's medical records, including a larger integration of physician-nurse communication around the study results and tracking the effects of participation on other medical co-morbidities may improve compliance and adherence. Prescribing minimal exercise and nutrition as medicine may have important benefits for disease prevention. Future larger exercise and nutrition trials implemented into a primary care clinical setting are encouraged.

## Data Availability Statement

Data sharing is not applicable for Phase I of this project. Deidentified data that supports the results from Phase II will be shared beginning 9 to 36 months following publication provided the investigator who proposes to use the data has approval from an Institutional Review Board (IRB), Independent Ethics Committee (IEC), or Research Ethics Board (REB), as applicable, and executes a data use/sharing agreement with UNC.

## Ethics Statement

The studies involving human participants were reviewed and approved by University of North Carolina Biomedical Institutional Review Board. The patients/participants provided their written informed consent to participate in this study.

## Author Contributions

All authors have met or exceeded the expectations of being an author according to the International Committee of Medical Journal Ethics. Specifically, AS-R developed the idea, designed and implemented the study, analyzed the results, and prepared the manuscript. MW assisted with the design of the study, analysis of the results and manuscript preparation. AV and MW helped develop and implement the study and assisted with manuscript preparation. MB and KH helped implement the study, analysis of the results, and manuscript preparation. All authors contributed to the article and approved the submitted version.

## Conflict of Interest

The authors declare that the research was conducted in the absence of any commercial or financial relationships that could be construed as a potential conflict of interest.

## Abbreviations

BMI: body mass index
CRF: cardiorespiratory fitness
CVD: cardiovascular disease
DBP: diastolic blood pressure
HR: heart rate
FMC: family medicine center
FM: fat mass
FFM: fat free mass
HDL: high density lipoproteins
HIIT: high intensity interval training
LDL: low density lipoproteins
PCMH: patient centered medial home
SBP: systolic blood pressure
TC: total cholesterol
TG: triglycerides
%Fat: percent body fat.
